# miR-496 inhibits proliferation via LYN and AKT pathway in gastric cancer

**DOI:** 10.1515/med-2021-0313

**Published:** 2021-08-25

**Authors:** Rui Su, Enhong Zhao, Jun Zhang

**Affiliations:** Department of Gastrointestinal Surgery, Affiliated Hospital of Chengde Medical College, 067000, Chengde, China; Department of General Surgery, Beijing Friendship Hospital, Capital Medical University, Beijing, China

**Keywords:** AKT/mTOR signaling pathway, apoptosis, binding site, miR-496, 3ʹ-UTR

## Abstract

MicroRNAs (miRNAs) operate as tumor suppressor or carcinogen to regulate cell proliferation, metastasis, invasion, differentiation, apoptosis, and metabolic process. In the present research, we investigated the effect and mechanism of miR-496 in human gastric cancer cells. miR-496 was downregulated in two gastric cancer cell lines, AGS and MKN45, compared with normal gastric epithelial cell line GES-1. miR-496 mimics inhibited the proliferation of AGS cells after the transfection for 48 and 72 h. The migration and invasion of AGS cells were also inhibited by the transfection of miR-496 mimics. miR-496 mimics induced the apoptosis through upregulating the levels of Bax and Active Caspase 3 and downregulating the levels of Bcl-2 and Total Caspase 3. Bioinformatics analysis showed that there was a binding site between miR-496 and Lyn kinase (LYN). miR-496 mimics could inhibit the expression of LYN in AGS cells. LYN overexpression blocked the inhibition of tumor cell growth, as well as the inhibition of AKT/mTOR signaling pathway induced by miR-496. In conclusion, miR-496 inhibited the proliferation through the AKT/mTOR signaling pathway via targeting LYN in gastric cancer cells. Our research provides a new potential target for clinical diagnosis and targeted treatment for gastric cancer.

## 1 Introduction

Gastric cancer is one of the most common human cancers, ranking third in the cancer-related deaths [1]. Studies show that both genetic and epigenetic factors are involved in the pathogenesis of human tumors, including gastric cancer [2]: however, due to the low sensitivity and specificity of tumor markers for gastric cancer, gastric cancer markers are relatively limited [3]. Therefore, the discovery of new markers and medicine for the diagnosis and treatment of tumor is an urgent problem [4,5]. miRNAs are non-coding RNAs with 21–25 bp [6]. They can reduce mRNA level by inhibiting mRNA translation or binding the 3ʹ-UTR region of mRNA, thus silencing their homologous target genes, playing an important role in cell processes and maintaining normal physiological environment [6]. Special miRNA operates as a tumor suppressor or carcinogen, so it is considered as a biomarker for early diagnosis and prognosis of cancer [7,8,9]. These miRNAs also regulate cell proliferation, metastasis, invasion, differentiation, apoptosis, and metabolic process [7,8,9].

miR-496 is an RNA gene and is affiliated with the miRNA class. It is located on human chromosome 14 and has unknown biological functions [10]. Limited studies have shown that low expression of miR-496 is related to aging process [11,12], and plays an important role in the formation and differentiation of bone cells [13]. miR-496 overexpression inhibits breast cancer cell proliferation through MBD2-dependence [14]. At present, there are few reports on miR-496, and its biological function in tumorigenesis is still unclear. In addition, miR-496 has not been reported in the proliferation and metastasis of gastric cancer cells. Therefore, we investigated the role and mechanism of miR-496 *in vitro* to provide new approach for clinical diagnosis and treatment of gastric cancer.

## 2 Materials and methods

### 2.1 Cell culture and transfection

Human normal gastric epithelial cell line GES-1 and gastric cancer cell lines AGS and MKN45 were all purchased from the cell bank of The Chinese Academy of Sciences (Shanghai, China). DMEM with 10% FBS was used for cell culture. miR-496 mimics, miRNA mimics negative control, and Lyn kinase (LYN) overexpression plasmid were purchased from GenePharma (Shanghai, China) and transfected into AGS cells using Lipofectamine 2000 (Invitrogen, CA, USA) according to the manufacturer’s protocol.

### 2.2 Fluorescence quantitative PCR (qPCR)

After the transfection for 24 h, total RNA was extracted from AGS cells by TRIzol reagent (Invitrogen, USA) and reverse transcripted to cDNA using M-MLV Reverse Transcriptase and TaqMan™ MicroRNA Reverse Transcription Kit (Invitrogen, USA). QPCR was performed to detect the level of miR-496 and LYN using the qPCR kit according to the manufacturer’s protocol (Invitrogen, USA). The relative expression of miR-496 and LYN was analyzed using 2^−ΔΔCt^ method.

### 2.3 Western blot

After the transfection for 48 h, whole-cell protein was extracted using a RIPA lysis buffer. The protein concentrations were detected by bicinchoninic acid method. Then, the protein extracts were separated using SDS-PAGE and transferred to a polyvinylidene fluoride membrane. After blocking with skimmed milk, the protein was incubated with the primary antibodies for 1 h, followed by secondary antibodies for 1 h. Primary antibodies, including anti-Bcl-2, anti-Bax, anti-total Caspase 3, anti-cleaved Caspase 3, anti-GAPDH, anti-Vimentin, anti-Cyclin D1, anti-Snail2, anti-p-AKT (Ser473), anti-p-mTOR (Ser2448), and anti-p70S6K (1:1,000), were purchased from Cell Signaling Technology Inc. (Danvers, MA, USA).

### 2.4 CCK8 assay

The proliferation of AGS cells was detected by Cell Counting Kit-8 (CCK8). After transfection, cells were transferred into a 96-well plate (1,000 cells/well). CCK8 reagent was added to the cells at a series of time points after the transfection (0, 24, 48, and 72 h). Cell viability was represented by OD value at 450 nm.

### 2.5 Clonogenic assay

After the transfection, about 500 cells were transferred into a 6 cm dish and cultured at 37°C for 2 weeks. Then, the colonies were fixed with methanol for 20 min and stained by 0.1% of crystal violet for 30 min. Visible colonies were counted separately by two researchers.

### 2.6 Transwell

For cell migration, AGS cells were transferred into the upper chamber of the transwell inserts after transfection (2 × 10^5^ cells). Serum-free medium was added into the lower chamber of the transwell inserts. After incubation for 48 h, AGS cells were fixed with methanol for 10 min and stained with 0.1% of crystal violet for 10 min. The migrated cells were counted in five random fields. For cell invasion, transwell inserts were pre-coated with Matrigel.

### 2.7 Flow cytometry analysis

After transfection for 48 h, the apoptosis of AGS cells was detected using Annexin-V and PI (BD Biosciences, Franklin Lakes, NJ, USA) following the manufacturer’s protocol. The percentage of apoptotic cells was analyzed on a FACScalibur flow cytometer using CellQuest Pro software (Becton, Dickinson and Company, USA).

### 2.8 Statistical analysis

A one-way analysis followed by post hoc Bonferroni test was performed to analyze the differences between groups. All data in the present study were analyzed and plotted with GraphPad Prism 8 (GraphPad Software Inc., San Diego, California, USA). All experiments were performed in triplicate. *P* < 0.05 was considered statistically significant.

## 3 Results

### 3.1 miR-496 inhibits the proliferation in gastric cancer cells

**Figure 1:**
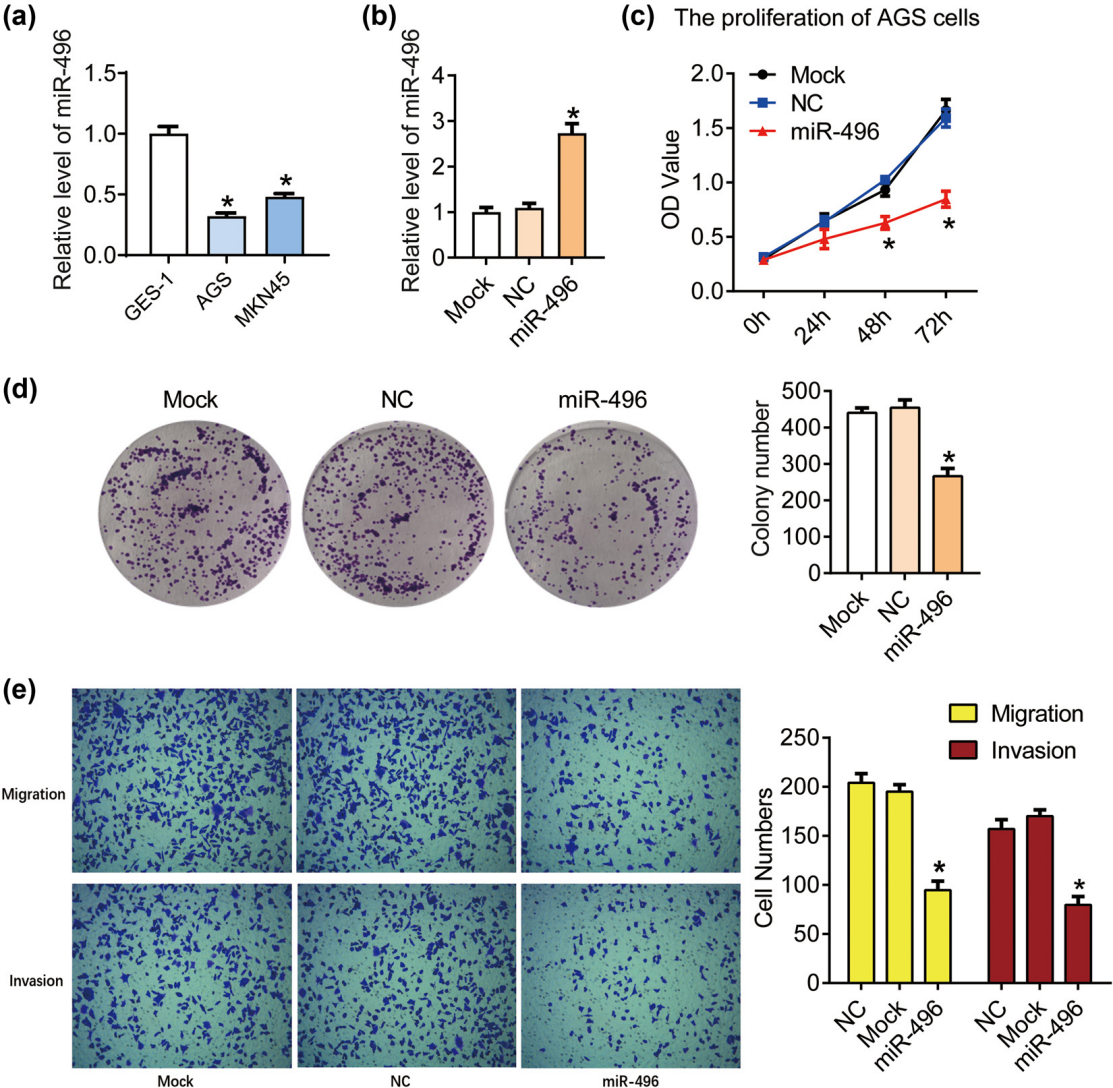
miR-496 inhibited the proliferation and metastasis in gastric cancer cells. (a) QPCR was performed to detect the level of miR-496 in AGS cells. (b) The level of miR-496 in AGS cells transfected with miR-496 mimics was detected by qPCR. Relative level of miR-496 was analyzed using 2^−ΔΔCt^ method and normalized to mock group. (c) The proliferation of AGS cells was determined using CCK8 assay. OD value (450 nm) was measured every 24 h. (d) Clonogenic assay was used to detect the proliferation of AGS cells. Colony number was counted 2 weeks after the culture. (e) The migration and invasion of AGS cells were detected by transwell assay after the transfection for 24 h. NC = negative control. *P < 0.05.

We first detected the expression of miR-496 in gastric cancer cell lines AGS and MKN45 by qPCR with the normal gastric epithelial cell line GES-1 as a control. miR-496 was downregulated in AGS and MKN45 compared with GES-1 cells (*P* < 0.05, Figure 1a). Then, the miR-496 mimics or miRNA mimics negative control was transfected into the AGS cells to generate miR-496 overexpressed (miR-496) or negative control (NC) cells, respectively. The level of miR-496 increased significantly in the miR-496 group compared with the NC group (*P* < 0.05, Figure 1b). The proliferation was detected using CCK8 and clonogenic assay. As shown in Figure 1c, the OD_450_ declined significantly after the transfection of miR-496 mimics for 48 and 72 h compared with the NC cells (*P* < 0.05). The colony numbers also markedly declined after transfection and incubated for 2 weeks compared with the NC cells (*P* < 0.05, Figure 1d). These data indicated that miR-496 could inhibit the proliferation of human gastric cancer cells.

### 3.2 miR-496 inhibits the migration and invasion in gastric cancer cells

Next the migration and invasion of the AGS cells with the transfection of miR-496 mimics were detected using transwell assay. As shown in Figure 1e, the migration cell numbers decreased after the transfection of miR-496 mimics (*P* < 0.05); the invasion cell numbers also declined after the transfection of miR-496 mimics compared with the miRNA mimics negative control (*P* < 0.05). These results suggested that miR-496 could inhibit the metastasis in gastric cancer cells.

### 3.3 miR-496 promotes the apoptosis in gastric cancer cells

**Figure 2:**
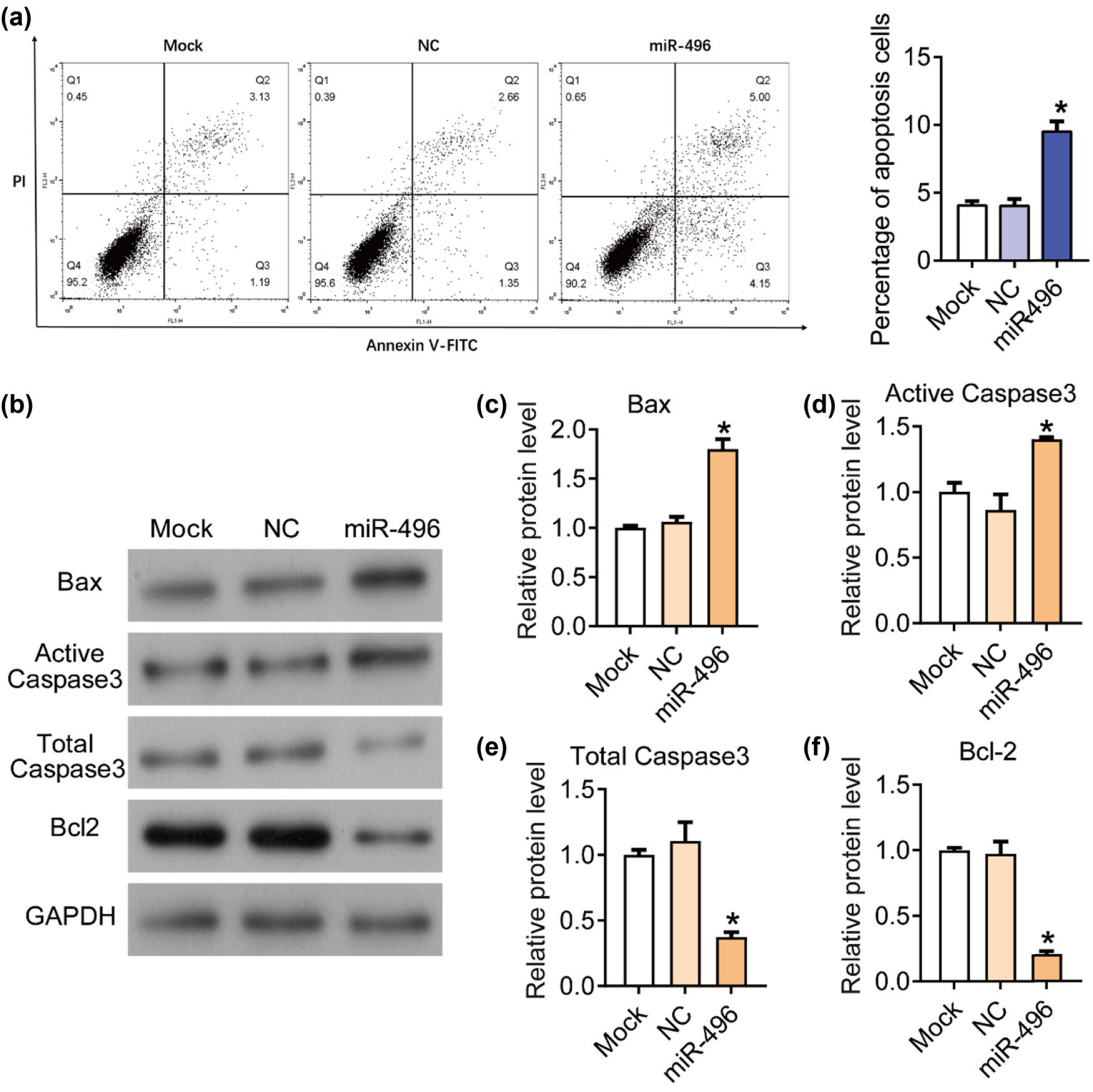
miR-496 promoted the apoptosis in gastric cancer cells. (a) Flow cytometry was performed to detect the apoptosis in AGS cells. (b) The expression levels of apoptosis-related proteins, (c) Bax, (d) active Caspase 3, (e) total Caspase 3, and (f) Bcl-2 were detected by western blot. The relative protein levels were normalized to mock group. NC = negative control. *P < 0.05.

Then, we detected the apoptosis of the AGS cells using flow cytometry. As shown in Figure 2a, the percentage of apoptosis cells in AGS cells transfected with miR-496 mimics (9.50 ± 0.75%) increased compared with the NC cells (4.03 ± 0.50%) (*P* < 0.05). In order to analyze the molecular mechanism by which miR-496 promotes tumor cell apoptosis, western blot was used to detect the expression of critical apoptotic factors in each group, including Bax, Active Caspase 3, Total Caspase 3, and Bcl-2. As shown in Figure 2b–f, miR-496 mimics upregulated the expression of Bax and Active Caspase 3, but downregulated the expression of Total Caspase 3 and Bcl-2 (*P* < 0.05). These results proved that miR-496 promoted the apoptosis through regulating apoptotic factors in gastric cancer cells.

### 3.4 miR-496 inhibits the expression of LYN in gastric cancer cells

**Figure 3:**
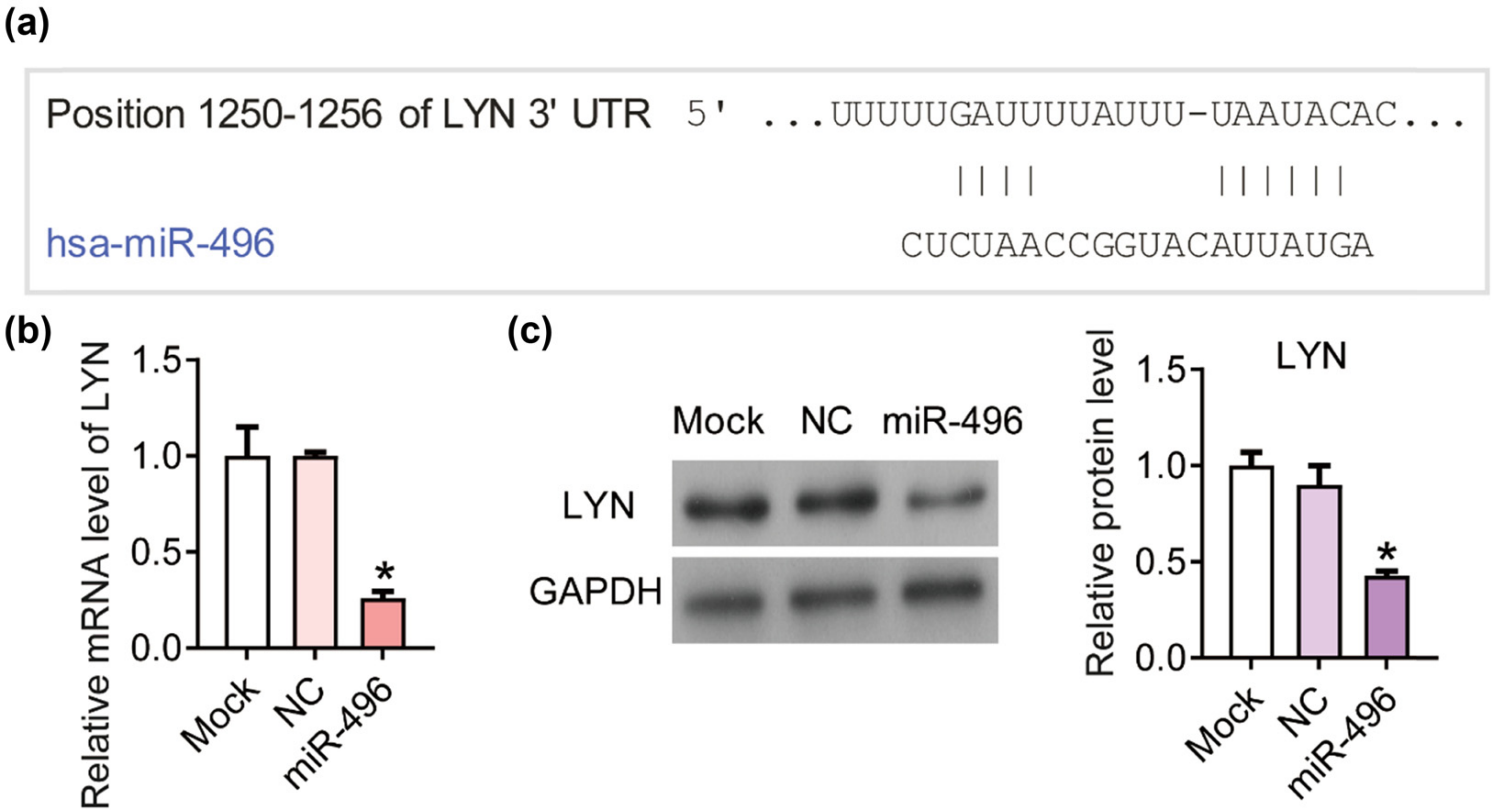
miR-496 inhibited the expression of LYN in gastric cancer cells. (a) According to the analysis on targetScan, there was a binding site between miR-496 and 3ʹ-UTR of LYN. (b) QPCR was performed to detect the level of LYN in AGS cells transfected with miR-496 mimics. Relative level of LYN was analyzed using 2^−ΔΔCt^ method and normalized to mock group. (c) The expression levels of LYN were detected by western blot. The relative protein levels were normalized to mock group. NC = negative control. *P < 0.05.

Next we predicted the potential binding targets of miR-496 using the online analysis tool TargetScan (http://www.targetscan.org/vert_71/) [15]. According to the analysis on targetScan, there was a binding site between miR-496 and 3ʹ-UTR of LYN (Figure 3a). After transfected with miR-496 mimics, the mRNA and protein level of LYN significantly declined compared with the NC cells according to the results of qPCR (Figure 3b) and western blot (Figure 3c). Thus, we hypothesized that LYN was the downstream direct target of miR-496 in gastric cancer cells.

### 3.5 LYN blocks the inhibition of tumor cell growth induced by miR-496 in gastric cancer cells

**Figure 4:**
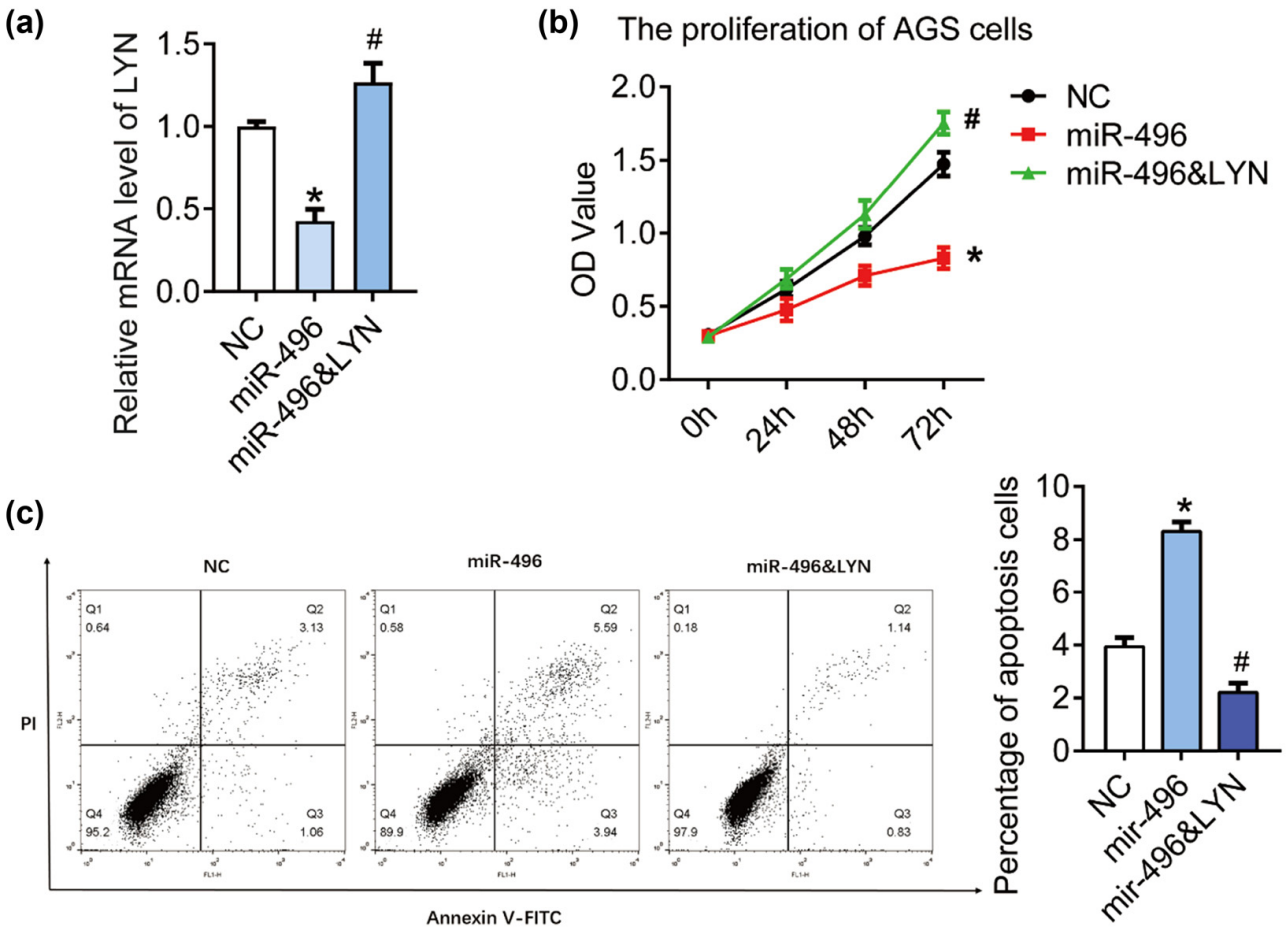
LYN blocked cell apoptosis induced by miR-496 in gastric cancer cells. (a) QPCR was performed to detect the level of LYN in AGS cells transfected with miR-496 mimics or miR-496 mimics + LYN overexpression. (b) The proliferation was detected by CCK8 assay. OD value (450 nm) was measured every 24 h. (c) Flow cytometry was performed to detect the apoptosis in AGS cells. NC = negative control. *P < 0.05; ^#^ *P* < 0.05.

In order to elucidate whether miR-496 exerts tumor suppressive effect in gastric cancer through LYN, we transfected miR-496 mimics and LYN overexpression plasmid (miR-496 & LYN) in AGS cell line at the same time (Figure 4a), and detected the cell proliferation and apoptosis with miR-496 mimics as a control. As shown in Figure 4b, OD_450_ of miR-496 & LYN group increased markedly compared with the miR-496 group (*P* < 0.05). The percentage of apoptosis cells in miR-496 & LYN group (2.20 ± 0.36%) declined compared with the miR-496 group (8.31 ± 0.35%) (Figure 4c, *P* < 0.05). These data indicated that miR-496 might inhibit the growth of gastric cancer cells by suppressing the expression of LYN.

### 3.6 miR-496 inhibits the AKT/mTOR signaling pathway via LYN in gastric cancer cells

Finally, we explored the molecular mechanism by which miR-496/LYN regulated the growth of gastric cancer. AKT/mTOR signaling pathway is widely involved in the origin and development of solid tumors and plays a key role in the proliferation and apoptosis of tumor cells [16,17]. In our previous studies, we found that the AKT/mTOR pathway was downregulated by LYN knockdown in AGS cells, including decreased levels of p-AKT, p-mTOR, and downstream effector p70. The AKT pathway activator IGF-1 could reverse the inhibitory effects of LYN knockdown on the proliferation, migration, and invasion in AGS cells.

**Figure 5:**
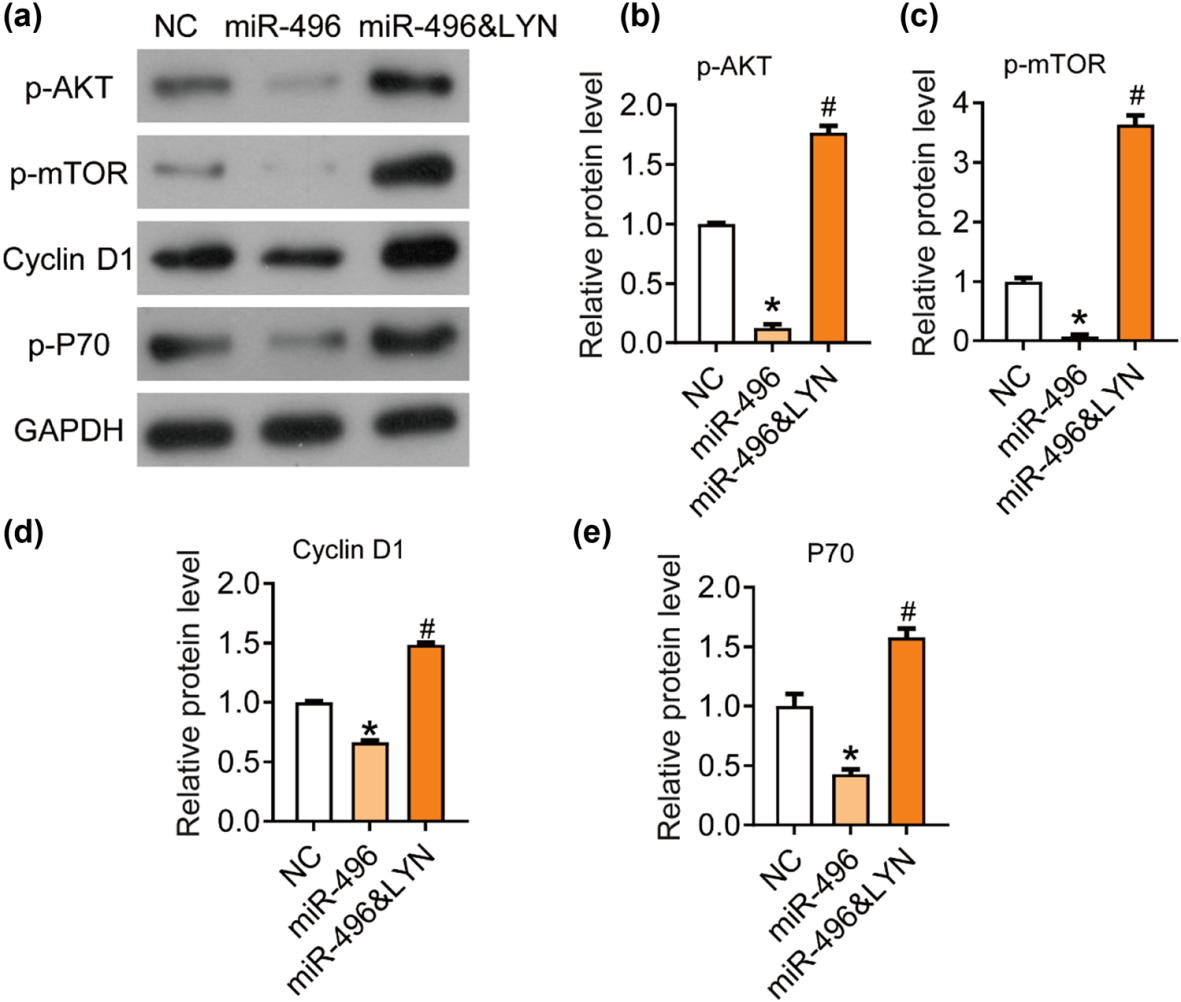
miR-496 inhibited the AKT/mTOR signaling pathway by targeting LYN in gastric cancer cells. (a) The expression levels of AKT/mTOR pathway-related proteins, (b) p-AKT, (c) p-mTOR, (d) Cyclin D1, (e) P70 were detected by western blot. The relative protein levels were normalized to NC group. NC = negative control. *P < 0.05; ^#^ *P* < 0.05.

Thus, in this research, we detected the expression of protein related to AKT/mTOR signaling pathway in each group using western blot (Figure 5a). The phosphorylation levels of AKT (Figure 5b) and mTOR (Figure 5c) and the levels of Cyclin D1 (Figure 5d) and P70 (Figure 5e) were significantly inhibited by the transfection of miR-496 mimics and rescued by the transfection of LYN overexpression plasmid. LYN overexpression blocked the inhibition of AKT/mTOR signaling pathway induced by miR-496 mimics. Out results indicated that miR-496/LYN inhibited the tumor growth by suppressing the AKT/mTOR signaling pathway.

## 4 Discussion

In recent years, the role of miR-496 in human tumors has been gradually revealed. lncRNA DANCR was found to be involved in the progression of lung cancer through sponging miR-496 and then regulating the level of mTOR [10]. miR-496 can also suppress gene expression and the proliferation in breast cancer cell lines MCF-10A, MCF-7, and MDA-MB-231 [14]. In another report, miR496 is downregulated by Human papillomavirus, notably type 16 and inhibit the post-transcriptional control of the transcription factor E2F2 in oropharyngeal cancer [18]. In the present study, we found that miR-496 was downregulated in AGS and MKN45 compared with the normal gastric epithelial cell line GES-1. At present, the expression of miR-496 in gastric cancer has not been reported. Our results suggest that miR-496 may play a role in the progression of gastric cancer, and is expected to be a clinical diagnostic indicator of gastric cancer, which still needs further experiments and clinical verification. Then, we proved that miR-496 could inhibit the proliferation, migration, and invasion, and induce the apoptosis in gastric cancer cells. Our data proved that miR-496 was involved in the progression of human gastric cancer.

Studies have shown that miRNA, which is combined with RNA-induced silencing complex, can recognize and bind 3ʹ-UTR of the target genes by incomplete or complete matching, and achieve post transcriptional regulation of target gene expression level [7,19,20]. In mammals, miRNA usually binds to the 3ʹ-UTR of the target gene through incomplete pairing, which affects the translation of target mRNA [20,21]. In addition, an miRNA can regulate multiple mRNA, and different miRNAs can also coordinate to regulate one mRNA molecule [20]. It is predicted that about one-third of the protein coding genes in the human cells are regulated by miRNA [22]. miRNAs and their target molecules form a complex regulatory network to control cell activity [22]. In the present research, we analyzed the potential target of miR-496 on targetScan. A binding site was found between miR-496 and the 3ʹ-UTR of LYN. QPCR result showed that miR-496 mimics significantly inhibited the expression of LYN in the AGS cells suggesting that LYN was a downstream target of miR-496 in gastric cancer. Then, we detected the proliferation and apoptosis in both miR-496 and LYN overexpressed cells with the only miR-496 overexpressed cells as a control. The results proved that LYN blocked the increase in cell proliferation and decrease in apoptosis induced by miR-496 in the AGS cells. LYN is a member of the Src family tyrosine kinases and operates as a pro-oncogene in the progression of human tumor [23,24]. Our previous studies showed that knockdown of LYN inhibited both proliferation and metastasis and resulted in the activation of the mitochondrial apoptotic pathway in AGS cells.

The progression of gastric cancer involves many signaling pathways [25,26,27,28,29]. Our previous study found that LYN can promote cell proliferation and metastasis in the AGS cells by activating AKT/mTOR signaling pathway. AKT/mTOR can regulate and stimulate cell growth by aggregating and integrating stimulating signals from nutrition, growth factors, energy, and environmental stresses to the cell, and is the hub of many important signal transduction pathways in the cell, involving in various biological functions such as gene transcription, protein translation, ribosome synthesis, and apoptosis [30]. Studies in recent years show that AKT/mTOR signaling pathway is closely related to the occurrence, development, and treatment of human tumors [31]. Therefore, we further studied the effect of miR-496/LYN on AKT/mTOR signaling pathway. miR-496 could significantly inhibit the phosphorylation of AKT and mTOR, as well as the expression of P70 and Cyclin D1, which were blocked by LYN overexpression. Therefore, we hypothesize that miR-496/LYN/AKT/P70 may be a cell growth regulatory pathway in gastric cancer cells. However, the role of miR-496 and LYN in gastric cancer still remains unclear and needs further exploration.

In conclusion, miR-496 inhibits the proliferation and metastasis and induces the apoptosis through targeting LYN and inhibiting the AKT/mTOR signaling pathway in gastric cancer. Our research provides a new potential target for clinical diagnosis and targeted treatment of gastric cancer.

## Abbreviations

LYN: Lyn kinase
miRNAs: microRNAs
CCK8: cell counting kit-8
NC: negative control
